# The Gür fibromyalgia model: a clinically operational framework for phenotype-informed, sequence-sensitive multimodal care

**DOI:** 10.3389/fpain.2026.1891033

**Published:** 2026-08-06

**Authors:** Ali Gür

**Affiliations:** Division of Algology, Department of Physical Medicine and Rehabilitation, Faculty of Medicine, Gaziantep University, Gaziantep, Türkiye

**Keywords:** chronic pain, fibromyalgia, gür fibromyalgia model, multimodal treatment, nociplastic pain, pain management, phenotyping, rehabilitation

## Abstract

Fibromyalgia affects approximately 2%–4% of adults and many patients obtain less than clinically meaningful benefit from any single pharmacological treatment, underscoring the need for a practical method of prioritizing multimodal care. This purposive narrative, hypothesis-generating review introduces the Gür Fibromyalgia Model (GFM), an expert-derived and currently unvalidated framework that organizes fibromyalgia around five interacting domains: central pain amplification, sleep dysregulation, load imbalance, peripheral drivers, and regulation deficit. Its therapeutic value lies not in introducing novel interventions but in sequencing familiar interventions with greater clinical deliberation. The model uses domain-weighted bedside assessment, phenotype-informed entry-point selection, and iterative reassessment to move from generic multimodal care toward deliberate, sequence-sensitive multimodal care. Tables 5, 6 provide an operationalization map and a provisional 0–3 research checklist with a defined recall window and decision rules. The GFM is not a replacement for diagnostic, nociplastic, biopsychosocial, clustering, or guideline frameworks; it is a testable sequencing hypothesis intended to determine whether closer alignment between dominant burden and the initial therapeutic lever improves adherence, tolerance, function, and early clinical trajectory. To our knowledge, the GFM is the first framework to operationalize domain-weighted, sequence-sensitive treatment entry-point selection as an explicitly testable clinical hypothesis within the constraints of standard fibromyalgia consultation.

## 1 Introduction

Fibromyalgia (FM) is a chronic pain syndrome defined by widespread pain, fatigue, non-restorative sleep, cognitive complaints, and variable functional limitation (1–8). Over the past decade, the adoption of symptom-based diagnostic criteria, the consolidation of the nociplastic pain construct, and broader acceptance of multimodal management have collectively strengthened the clinical legitimacy of FM and brought greater clarity to its treatment landscape (1–8). Nonetheless, a fundamental problem persists in routine practice: patients who fulfill the same diagnostic criteria may differ markedly in the symptoms that dominate their clinical picture, the factors that precipitate worsening, and the treatments they are able to tolerate or maintain over time.

FM is common and costly: population estimates generally fall near 2%–4%, while the syndrome generates substantial direct and indirect societal costs (1, 9, 10). Average treatment effects also conceal considerable non-response; across commonly used medicines, only a minority achieve substantial pain relief, and many patients fail to reach a 30% reduction in pain (11–13). This therapeutic variability is consistent with cluster research showing clinically distinct subgroup patterns rather than a uniform presentation (14, 15).

In some patients, disordered sleep occupies the center of the clinical presentation. In others, the principal limitation is load intolerance, persistent peripheral pain generators, or pronounced autonomic-affective instability. Although this heterogeneity is widely acknowledged in the literature, it has been less consistently translated into a practical bedside structure that helps clinicians determine what to address first, how to pace the therapeutic sequence, and when to recalibrate the treatment plan.

The present review introduces the Gür Fibromyalgia Model (GFM) as a pragmatic response to that gap. Existing approaches include symptom-severity continua, OMERACT outcome domains, data-driven clusters, nociplastic and mixed-pain phenotyping, and the stepwise EULAR management framework (1, 2, 14–17). These approaches are essential for diagnosis, mechanism description, outcome measurement, or broad treatment selection, but they do not consistently provide a bedside rule for choosing the first therapeutic lever when several guideline-concordant options are available. The GFM does not seek to replace them. It organizes FM into five interacting domains—central pain amplification, sleep dysregulation, load imbalance, peripheral drivers, and regulation deficit—and links current domain weight to provisional treatment sequencing and prospective hypothesis testing.

## 2 Methods: narrative literature review

This article was conceived as a purposive narrative conceptual review and model paper, not as a systematic or scoping review. PubMed/MEDLINE, Scopus, and Google Scholar were searched iteratively for clinical practice guidelines, landmark mechanistic studies, systematic reviews, meta-analyses, cluster studies, measurement papers, and clinically relevant trials addressing FM diagnosis, nociplastic pain, sleep disturbance, autonomic function, peripheral pain contributors, exercise and rehabilitation, self-management, pharmacotherapy, and phenotyping. Priority was given to influential work published between 2016 and 2026, while earlier landmark publications—including foundational studies on sleep and pain (18, 19), fatigue in fibromyalgia (20), and neurobiological features of central sensitization (21)—were retained for conceptual continuity. References were selected for their relevance to model construction and clinical operationalization rather than through exhaustive screening or formal risk-of-bias grading. Consequently, the synthesis is inherently vulnerable to selection and confirmation bias, especially because the proposed model may influence which evidence appears most salient. The review should therefore be read as a transparent hypothesis-generating synthesis whose propositions require independent systematic review and prospective validation.

## 3 Rationale for the Gür fibromyalgia model

A new clinical model can only be justified if recurrent problems in patient care remain unresolved despite existing frameworks. In FM, the first such problem is the gap between diagnosis and treatment stratification. The 2016 ACR revision substantially improves case identification, yet it does not indicate which pathophysiological mechanisms predominate in a given patient or which treatment should be prioritized as the initial intervention (1).

The second problem is the gap between mechanistic sophistication and bedside usability. The contemporary FM literature addresses altered central pain processing, sleep architecture disruption, autonomic dysfunction, small-fiber pathology in a subset of patients, neuroimmune changes, and psychosocial burden, yet these insights have not always been distilled into a compact clinical framework that can be applied during a routine consultation (5–8, 22–25). Clinicians still lack a model that can hold pain, sleep, load tolerance, peripheral nociceptive input, and regulatory problems within a single decision structure.

The third problem is the gap between multimodality and sequencing. Current guidelines endorse multimodal care, but they offer comparatively little guidance on entry-point logic: when sleep stabilization should precede rehabilitation, when peripheral drivers warrant explicit treatment, and when regulation problems may undermine otherwise appropriate interventions (2, 4, 26–31). The GFM addresses these gaps by asking not only what belongs in FM care, but what should come first, at what intensity, and on what clinical grounds.

## 4 Conceptual architecture of the GFM

The GFM is a clinically oriented framework rather than a new diagnostic system or a single-cause theory. Its five domains were chosen pragmatically because they represent recurring treatment-relevant problems that can alter the timing, tolerability, or likely yield of familiar interventions: central pain amplification, sleep dysregulation, load imbalance, peripheral drivers, and regulation deficit. The number and boundaries were not derived through factor analysis, Delphi consensus, or data-driven clustering. Five domains should therefore be viewed as a parsimonious working architecture to be challenged empirically, not as a claim that FM possesses exactly five natural biological subtypes.

These domains are neither mutually exclusive nor equally weighted across patients. They are best understood as interacting influences whose relative importance shifts over time. The decision to organize FM around five domains is therefore pragmatic: the number is broad enough to reflect the syndrome's genuine complexity, yet compact enough to remain usable at the bedside within the constraints of a standard consultation.

Several clinically important features are intentionally nested within existing domains rather than treated as separate categories. Cognitive complaints, for instance, are distributed across sleep dysregulation, regulation deficit, and central amplification. Immune and neuroinflammatory findings inform mechanistic understanding but do not yet function as stand-alone bedside domains amenable to direct therapeutic targeting. Regional musculoskeletal findings become relevant within the model when they act as peripheral drivers in the context of a confirmed nociplastic presentation (22–25).

The term regulation deficit is used with deliberate intent. It refers to instability in restoring physiological, behavioral, and symptom equilibrium after ordinary perturbations. In this context, regulation is conceived more broadly than dysregulation of any single system, yet it remains clinically anchored: the central question is how effectively the patient re-establishes a workable baseline after physical, emotional, cognitive, or sleep-related strain. The term therefore emphasizes dynamic recovery failure rather than a fixed psychiatric or metaphysical label.

For bedside separation of overlapping domains, the GFM applies a proximal-target rule. Sleep dysregulation is weighted when the primary abnormality is sleep initiation, continuity, timing, non-restorative sleep, or next-day worsening directly linked to sleep. Load imbalance is weighted when symptoms track the quantity, pattern, or timing of physical, cognitive, occupational, or social demand. Regulation deficit is weighted when the defining feature is disproportionate physiological or behavioral reactivity and delayed return to baseline across more than one type of perturbation, not merely after exertion or a poor night of sleep. Co-dominance is expected. When two domains receive equal high scores, the initial entry point is selected by safety, reversibility, patient preference, treatment burden, and the probability that one domain blocks engagement with the other; sleep disorders and modifiable peripheral pathology are addressed before escalating rehabilitation when clinically indicated. These provisional tie-breakers are operational aids, not validated decision rules.

**Figure 1:**
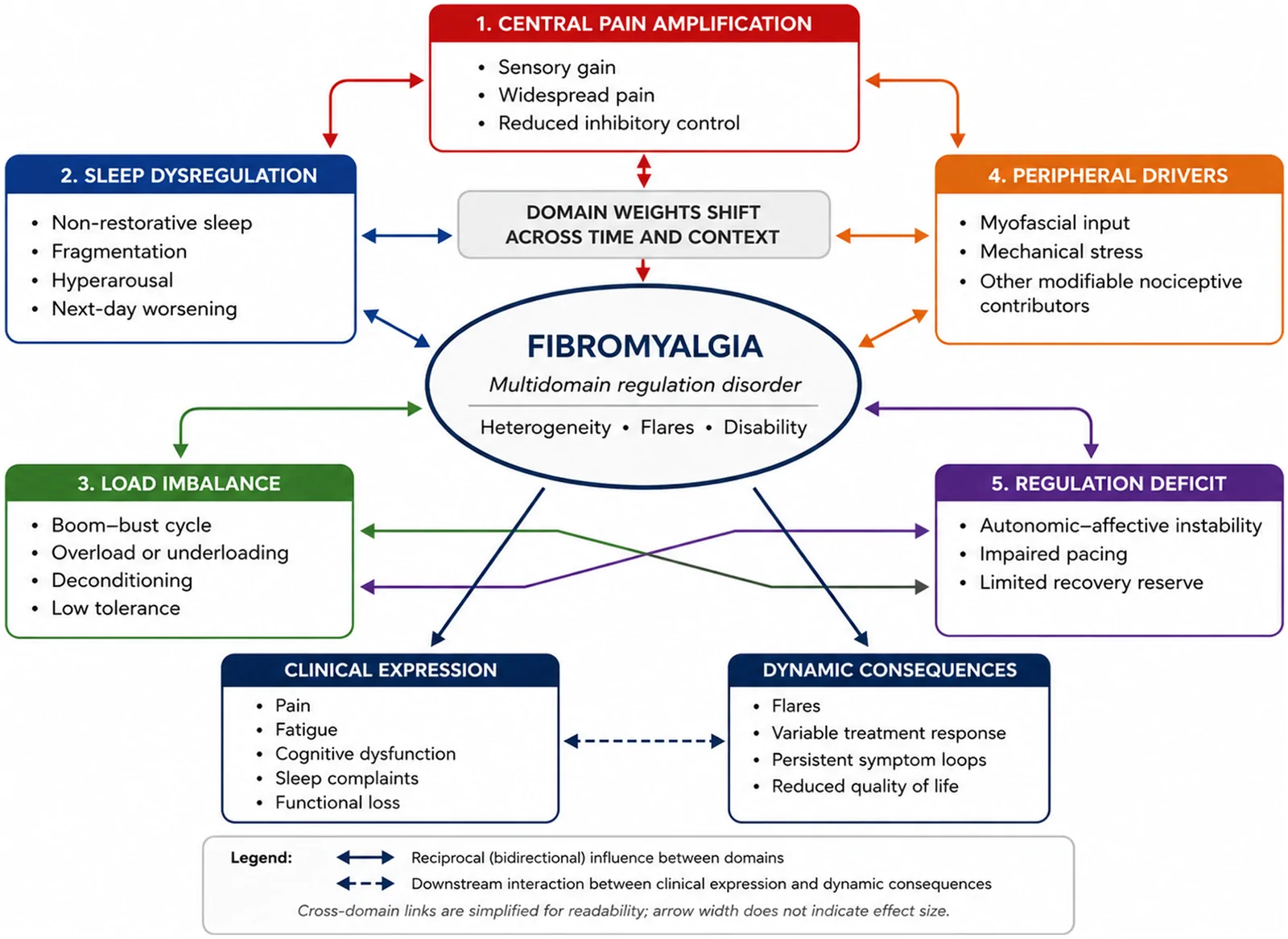
The Gür fibromyalgia model conceptualizes fibromyalgia as a multidomain regulation disorder shaped by five interacting domains. Bidirectional arrows denote proposed reciprocal influence rather than established causal effect sizes. Cross-domain links are simplified for readability. The model is a hypothesis-generating clinical architecture, not a validated biological network.

Figure 1 summarizes the five-domain structure and its principal feedback loops.

## 5 The five core domains

### 5.1 Central pain amplification

Central pain amplification is indispensable to any serious model of FM. It captures altered nociceptive processing, sensory gain, and the mismatch between symptom intensity and identifiable structural injury, helping to explain why FM pain is real despite the absence of a single anatomical lesion (3, 32–34). Within the GFM, however, central amplification is treated as one major domain among several rather than as the entirety of the syndrome. This distinction carries direct clinical implications, because patients with comparable levels of amplification may nevertheless differ substantially in sleep stability, load tolerance, peripheral nociceptive input, and regulatory capacity.

### 5.2 Sleep dysregulation

Sleep dysregulation encompasses non-restorative sleep, insomnia, circadian irregularity, poor sleep continuity, and clinically relevant morning worsening. In a substantial proportion of patients, sleep is not merely an associated symptom but a threshold domain that shapes pain severity, fatigue, cognitive function, and next-day treatment tolerance (18, 19, 29, 35, 36). Within the GFM, a sleep-dominant presentation often justifies earlier stabilization of sleep before rehabilitation intensity is escalated.

### 5.3 Load imbalance

Load imbalance refers to a mismatch between total demand and recovery capacity. Load is defined broadly in this context and includes physical, cognitive, occupational, emotional, and social demands. The domain encompasses both overload and underloading, including boom-and-bust pacing patterns, post-exertional worsening, deconditioning, and poor tolerance of generic exercise prescriptions (26, 27, 31). The therapeutic implication is recalibration of the load–recovery ratio rather than simple encouragement to increase activity.

### 5.4 Peripheral drivers

Peripheral drivers are local or regional nociceptive contributors that may amplify or sustain overall FM burden. Examples include active myofascial pain, osteoarthritis, obesity-related biomechanical stress, hypermobility-related strain, and sleep-related peripheral discomfort. These contributors alone are not sufficient to explain the full FM syndrome, nor should their identification displace a confirmed nociplastic context. Their treatment is biologically plausible and clinically reasonable, but direct randomized evidence that treating a peripheral driver improves global FM outcomes remains limited. The domain should therefore be interpreted as a modifiable-input hypothesis rather than a proven route to syndrome-wide improvement.

### 5.5 Regulation deficit

Regulation deficit refers provisionally to impaired restoration of autonomic, behavioral, and symptom equilibrium after ordinary perturbations. The construct is anchored in recovery dynamics rather than in a psychiatric label: a patient may show disproportionate tachycardia, orthostatic symptoms, stress reactivity, sleep reactivity, symptom volatility, or prolonged return to baseline after modest physical, emotional, or cognitive demand. Fibromyalgia studies have repeatedly reported altered autonomic profiles, including reduced heart-rate variability and abnormal sympathetic-parasympathetic balance, although direction, magnitude, protocols, and clinical specificity vary across studies (24, 37–39). Candidate physiological measures include resting and challenge-related RMSSD, frequency-domain HRV indices, orthostatic heart-rate and blood-pressure responses, and—in research settings—cortisol awakening response or sympathetic skin response. None is sufficiently specific to diagnose a GFM domain, and LF/HF should not be treated as a simple measure of sympathovagal balance. Bedside corroboration should therefore combine physiology with validated or structured patient-reported measures, such as orthostatic symptom history, Perceived Stress Scale, symptom-reactivity and recovery-time diaries, and consistency of daily routines. The concept is also compatible with allostatic-load theory, in which repeated adaptive demands can produce cumulative regulatory cost and slower recovery (40). Operationally, regulation deficit is distinguished from load imbalance when delayed recovery generalizes across multiple perturbation types rather than tracking mainly the amount or pattern of activity; it is distinguished from sleep dysregulation when instability persists independently of a primary sleep-timing or sleep-continuity disorder. These distinctions are provisional. HRV and other biomarkers are proposed as research correlates, not validated diagnostic tests or mandatory treatment selectors.

## 6 Dynamic interactions and phenotypic presentations

The full clinical value of the GFM emerges when the domains are treated as a dynamic network rather than as a static list. Experimental and longitudinal evidence supports bidirectional links between sleep disturbance and pain amplification (41), while fear, catastrophizing, and avoidance can reduce activity tolerance and reinforce disability (42). Regional nociceptive input may augment widespread hypersensitivity in susceptible patients, although the degree to which local treatment changes global FM outcomes remains uncertain (43). Repeated physiological and behavioral strain may also slow recovery through autonomic and allostatic mechanisms (24, 37–40). Figure 1 depicts these proposed pathways as reciprocal influences rather than proven causal coefficients.

This network logic explains why patients who carry the same diagnosis may require markedly different treatment sequences. Clinically recognizable patterns include amplification-dominant, sleep-dominant, load-imbalance, peripheral-driver, regulation-deficit, and high-impact mixed presentations. These are working phenotypes rather than fixed categories; Table 1 provides brief bedside vignettes to illustrate each pattern.

**Table 1:** Illustrative phenotype vignettes supporting bedside use of the Gür fibromyalgia model.

Phenotype	Illustrative vignette
Amplification-dominant	A 44-year-old patient has diffuse allodynia, sensory hypersensitivity, and widespread pain without a major sleep disturbance or dominant regional pain generator. Education, graded sensory exposure, and tolerance-based rehabilitation are the initial priorities. Empirical context: (14, 34).
Sleep-dominant	A 41-year-old woman reports waking unrefreshed every morning, marked morning stiffness, cognitive fog, and disproportionate pain escalation after even minor sleep disruption. Previous rehabilitation attempts repeatedly fail after poor-sleep nights. Empirical context: (15, 18, 19, 35, 36).
Load-imbalance	A 46-year-old teacher alternates between overactivity on “good days” and several days of post-exertional worsening, with declining confidence in movement and progressive deconditioning. Symptoms are strongly linked to mismatch between demand and recovery. Empirical context: (14, 15, 26, 27).
Peripheral-driver	A 52-year-old woman meets fibromyalgia criteria but also has active trapezial and gluteal myofascial pain, obesity-related biomechanical strain, and knee osteoarthritis. Generalized symptoms improve only partially unless these local drivers are addressed. Empirical context: (14, 16, 22).
Regulation-deficit	A 38-year-old patient shows marked stress reactivity, palpitations, symptom volatility, erratic routines, and prolonged recovery after minor perturbations despite no major inflammatory or structural explanation. The dominant problem is unstable regulation rather than pain intensity alone. Empirical context: (14, 24, 37–40).
High-impact mixed	A 49-year-old patient presents with severe sleep disturbance, diffuse pain amplification, pacing failure, and meaningful regional pain generators simultaneously. The clinical task is to identify the safest and highest-yield entry point without overloading the patient with simultaneous interventions. Empirical context: (14, 15).

GFM, Gür fibromyalgia model.

Because domain dominance can shift over time, reassessment should be built into the care plan. A practical rhythm is every four to eight weeks, and sooner after a major flare, a medication change, a newly recognized comorbidity, or failure of the initial treatment entry point.

## 7 Therapeutic translation of the GFM

The therapeutic value of the GFM lies not in introducing novel interventions but in sequencing familiar ones with greater clinical deliberation. The practical question at each stage is which low-burden intervention is most likely to reduce the dominant barrier to participation or recovery. This sequencing proposition is clinically plausible but remains untested.

### 7.1 Exercise and rehabilitation

Exercise remains a central component of FM management, and systematic reviews support resistance and home-based exercise programs (26, 27). The GFM proposes—without claiming direct comparative evidence—that starting intensity and progression should be adjusted to current load tolerance, sleep stability, and recovery capacity. No trial has yet shown that GFM-matched rehabilitation is superior to standard, guideline-concordant exercise. This is therefore a prospective treatment-matching hypothesis: low-burden, tolerance-based progression may improve adherence and reduce early withdrawal in patients whose initial barrier is poor sleep, boom-and-bust activity, or prolonged recovery.

### 7.2 Sleep as a treatment-readiness domain

Sleep may warrant earlier prioritization than it typically receives in current practice. In sleep-dominant phenotypes, partial restoration of sleep quality can improve next-day tolerance for pacing and rehabilitation. This approach does not represent therapeutic passivity; it reflects the recognition that sleep can function as a treatment-readiness domain whose stabilization enables downstream therapeutic engagement (29, 35, 36).

### 7.3 Pharmacotherapy within a sequencing logic

Pharmacotherapy can be integrated within the same sequencing logic, but drug-domain matching should be distinguished from established efficacy. Evidence supports modest average benefits for duloxetine, pregabalin, and low-dose amitriptyline, with substantial non-response and adverse-event discontinuation (11–13, 44). Milnacipran is another approved option in some jurisdictions; tramadol has limited evidence and opioid-related risks; and low-dose naltrexone remains investigational despite encouraging early studies. Bedtime sublingual cyclobenzaprine has phase 3 evidence for pain, sleep, fatigue, and function and may be relevant when non-restorative sleep is prominent (36). Statements that a particular drug is especially suitable for a GFM phenotype are hypotheses unless supported by phenotype-stratified trials. Medication selection must continue to follow local approvals, contraindications, comorbidity, adverse-effect profiles, patient preference, and guideline recommendations.

### 7.4 Education and self-management

Education and self-management constitute therapeutic infrastructure rather than ancillary additions. They should validate the patient's pain experience, communicate the multidomain logic of the syndrome, reduce false structural alarm, and support pacing, activity planning, and sustainable coping strategies (30, 31).

### 7.5 Psychologically informed interventions

Psychologically informed interventions fit the model through a regulation lens. Cognitive-behavioral and acceptance-based approaches can help patients reduce symptom-related fear, improve pacing decisions, and stabilize daily routines (28, 45). Their role within the GFM is not to psychologize FM but to strengthen recovery capacity when regulation problems are clinically prominent.

### 7.6 Peripheral drivers

Peripheral drivers should be treated pragmatically but without peripheralizing the syndrome. Local rehabilitation, biomechanical correction, weight-sensitive load management, and selected trigger-point or regional interventions may reduce background nociceptive load and thereby render global care more effective (4–6, 22, 23).

**Figure 2:**
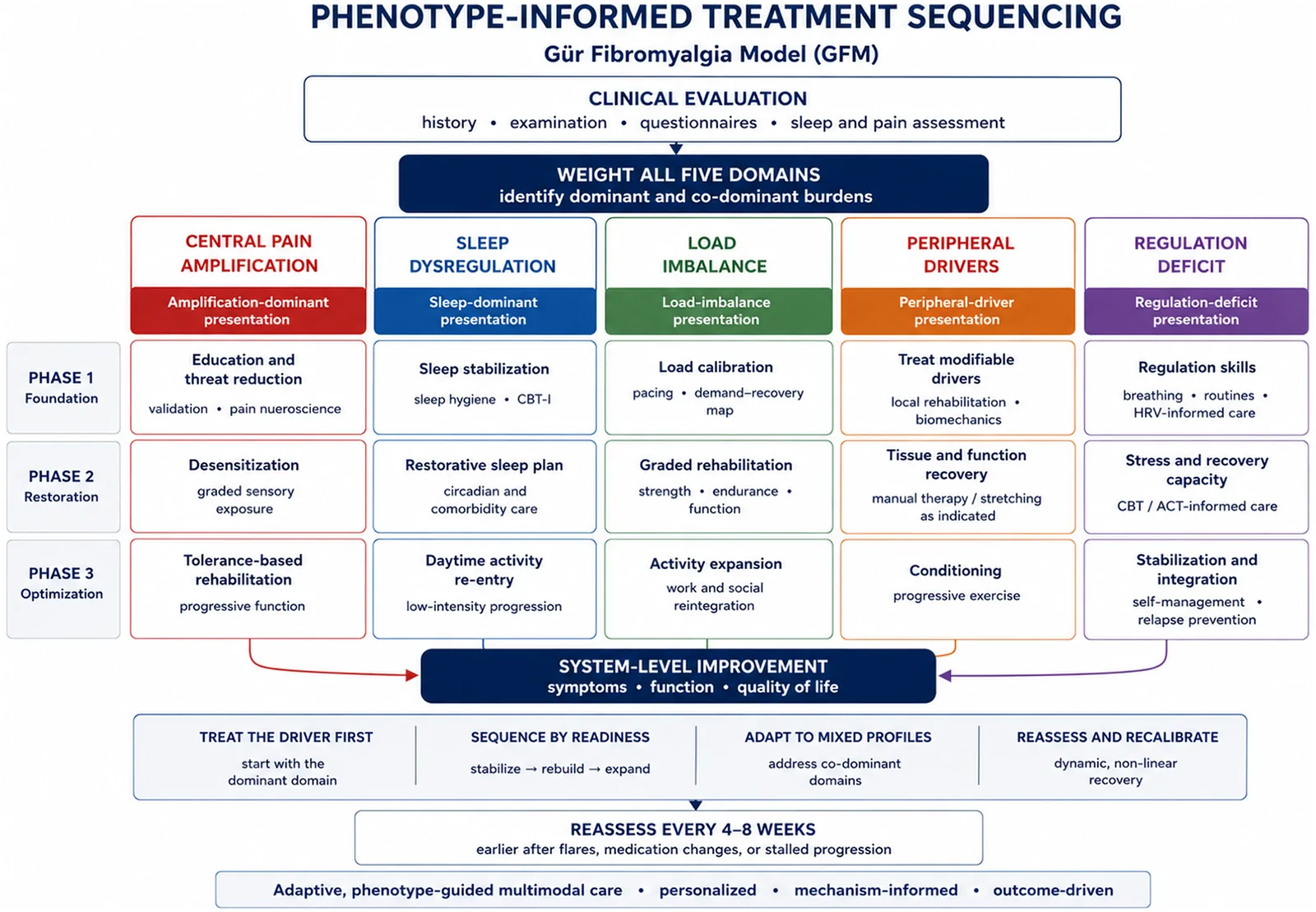
Phenotype-informed treatment sequencing in the Gür fibromyalgia model. All five domains are weighted over the previous 2 weeks; co-dominant profiles are resolved provisionally using safety, reversibility, treatment-readiness, burden, and patient preference. The three phases represent foundation, restoration, and optimization, with reassessment every 4–8 weeks or earlier after flares, medication changes, adverse effects, or stalled progression.

The net result is a shift from generic multimodal care to more deliberate, sequence-sensitive multimodal care. Figure 2 presents a phenotype-informed treatment sequence derived from this logic, and Table 2 summarizes a practical bedside workflow for applying the model in clinical practice.

**Table 2:** Bedside application of the Gür fibromyalgia model.

Step	Clinical action and decision rule
0	Establish baseline severity and function with the FIQR and record current medications, sleep disorders, comorbidities, red flags, and patient priorities.
1	Confirm fibromyalgia using accepted criteria and exclude active alternative explanations that would redirect management.
2	Rate all five domains over the previous 2 weeks using Table 6; a score of 3 identifies a candidate dominant domain, while equal scores of 2–3 indicate co-dominance.
3	Separate overlaps using the proximal-target rule: sleep timing/continuity → sleep; demand-pattern mismatch → load; cross-perturbation reactivity/delayed recovery → regulation.
4	If a single domain has emerged from Step 3, proceed directly to Step 5. If two or more domains remain co-dominant after overlap resolution, apply tie-breakers in order: urgent safety/comorbidity, reversible sleep or peripheral pathology, treatment that unlocks participation, lower burden/risk, and patient preference.
5	Start one principal low-burden intervention and document the hypothesized mechanism and expected early signal; do not initiate the full multimodal package at maximum intensity.
6	Reassess at 4–8 weeks (earlier after a flare, medication change, adverse effect, or deterioration) using FIQR plus domain-specific measures.
7	Continue, progress, combine, or recalibrate according to benefit, tolerance, and any shift in domain weight; lack of response should trigger reconsideration of the entry-point hypothesis and differential diagnosis.

## 8 Discussion

The GFM is intended to address a narrow clinical problem: how to translate acknowledged heterogeneity into an initial treatment sequence. It should not be read as a hierarchy in which established frameworks are displaced. Diagnostic criteria, nociplastic pain theory, biopsychosocial formulations, mixed-pain phenotyping, and data-driven clusters answer different questions and are generally supported by a larger empirical literature than the GFM.

The point of divergence is functional rather than etiologic. The biopsychosocial model provides broad contextual and causal coverage, whereas the GFM compresses selected treatment-relevant features into domains that can be weighted and prioritized. Nociplastic and central-sensitization frameworks place altered central processing at the main explanator*y* axis, whereas the GFM treats central amplification as one interacting domain. Mixed-pain frameworks classify pain mechanisms, whereas the GFM treats peripheral nociceptive input as a modifier within a confirmed nociplastic presentation and adds non-pain domains that may determine treatment readiness.

Data-driven cluster models have a clear empirical advantage: subgroup solutions are generated from observed patient data rather than expert synthesis (14). Their limitations are different from those of the GFM: cluster number and composition vary with the variables, sample, and analytic method, and cluster membership does not necessarily provide a bedside rule for which intervention should be started first. The GFM should therefore be tested against, not positioned above, data-driven clusters; concordance, discordance, and comparative prediction of treatment response are important validation questions.

The model also has partial, purpose-dependent alignment with the OMERACT fibromyalgia core domain set, which was developed to standardize outcome assessment rather than treatment sequencing (17). Pain and tenderness overlap most closely with central amplification and peripheral drivers; sleep disturbance maps directly to sleep dysregulation; fatigue and multidimensional function are partly represented through load imbalance and system-level outcomes; and patient global response, cognitive dysfunction, and depression are better understood as cross-domain consequences or modifiers than as one-to-one GFM domains. This approximate mapping provides a route to construct validation without implying that the two frameworks are interchangeable.

The GFM is also intended to complement, not replace, the EULAR stepwise approach (2). EULAR appropriately begins with education and non-pharmacological management and then tailors options to pain, sleep, mood, and disability. The narrower GFM proposition is that, even within this guideline-concordant menu, explicitly weighting the current dominant and co-dominant barriers may help clinicians choose order, initial intensity, and timing of reassessment. Whether this produces better outcomes than usual stepwise care is unknown and should be tested directly.

The five-domain architecture is intended as a pragmatic clinical tool. Cognitive complaints, for example, are not separated into an independent domain because they typically reflect overlapping influences from poor sleep, central amplification, and impaired regulation. Similarly, immune and neuroinflammatory findings are mechanistically important, but the existing evidence does not yet justify treating them as a stand-alone bedside domain for the purpose of treatment sequencing (22, 23).

Sleep merits particular emphasis within this framework. Non-restorative sleep can precede and amplify pain, fatigue, cognitive dysfunction, and functional impairment, while chronic pain can itself worsen sleep, creating a bidirectional loop (35, 36, 41). In a sleep-dominant presentation, sleep stabilization may function as a treatment-readiness intervention rather than a secondary comfort measure. The stronger claim—that sleep treatment is a prerequisite for effective rehabilitation—remains a testable hypothesis and should not be generalized to all patients.

The same logic applies to regulation deficit. Framed as a systems concept rather than a psychiatric label, it draws attention to the patient's capacity to recover equilibrium after ordinary perturbations. This perspective helps to explain why otherwise well-designed treatment plans may fail when pacing, recovery, and autonomic stability are insufficiently supported (24, 28, 45).

The model also speaks to the fragmentation of care that many FM patients experience. Patients often move between specialties and require recalibration as symptom priorities shift (46, 47). The rheumatology referral-based, general-internal-medicine-led clinic described by Pressimone and colleagues provides a relevant implementation precedent: structured assessment, education, coordinated management, and follow-up were feasible and associated with preliminary patient improvements (47). That service was not a validation of the GFM, but it illustrates how a consistent clinical pathway can reduce fragmentation and offers a practical setting in which domain weighting and sequencing could be prospectively evaluated.

Pharmacological and non-pharmacological strategies fit naturally within this framework when they are deployed to lower the most dominant barrier rather than being added indiscriminately. In that sense, the model is less about expanding the treatment menu than about improving clinical order (48).

A suitable first empirical test would be a prospective cohort study in which patients undergo baseline domain weighting followed by short-term phenotype-sensitive treatment allocation, with reassessment of symptom burden, functional status, adherence, and treatment tolerance over time. Such a design would test the model where it matters most: not simply in descriptive accuracy, but in whether domain-treatment matching improves therapeutic fit and early clinical trajectory. Tables 3, 4 present the complementary strengths, evidence status, and sequencing limitations of major frameworks rather than a superiority hierarchy.

**Table 3:** Comparative positioning of the Gür fibromyalgia model alongside major conceptual frameworks in fibromyalgia.

Model/framework	Primary focus	Established strengths/evidence status	Limits for sequence-sensitive care	Relationship to the GFM	Key refs
2016 ACR criteria	Positive symptom-based diagnosis using generalized pain and symptom severity.	Widely used diagnostic framework with established validity; improves case definition and reduces reliance on tender-point examination.	Identifies who has FM but does not determine the dominant current burden or treatment order.	The GFM begins after diagnosis and does not replace the criteria.	(1)
Nociplastic/central-sensitization model	Altered nociceptive processing and central sensory amplification.	Supported by an extensive mechanistic and clinical literature; validates symptoms and informs education and centrally directed care.	Does not by itself specify whether sleep, load, peripheral input, or regulation is the best initial leverage point.	The GFM retains amplification as one domain; it is clinically narrower and empirically less mature.	(3, 33)
Biopsychosocial model	Reciprocal biological, psychological, behavioral, and social influences.	Well-established, comprehensive, and useful for therapeutic alliance and contextual formulation.	Its breadth does not automatically provide domain weighting or an explicit treatment sequence.	The GFM operationalizes selected treatment-relevant features into a narrower, unvalidated prioritization scheme.	(51)
Stress-system/stress-disorder model	Stress responsivity, autonomic reactivity, and impaired recovery.	Explains symptom volatility and prolonged worsening in patients with prominent stress and autonomic features.	May over-center stress and fit less well when sleep, load, or peripheral drivers dominate.	Regulation deficit draws from this tradition without treating stress as a universal primary mechanism.	(24, 52)
Integrative neurophysiological–psychosocial model	Dynamic crosstalk between neurophysiological and psychosocial processes.	Offers strong systems-level synthesis and preserves mechanistic complexity.	Can remain too abstract for rapid bedside prioritization.	The GFM offers a simpler operational translation but necessarily loses mechanistic granularity.	(32)
Mixed-pain/pain-phenotyping model	Nociplastic pain with possible nociceptive and/or neuropathic components.	Supports mechanism-specific pain phenotyping and recognition of clinically meaningful peripheral contribution.	Primarily classifies pain mechanisms and gives less guidance on non-pain treatment readiness.	Peripheral drivers are modifiers within the GFM, not a synonym for a mixed-pain phenotype; sleep, load, and regulation are assessed separately.	(16, 22)
Data-driven cluster models	Empirical patient subgroups derived from multivariable symptom or mechanism data.	Empirically generated and able to identify heterogeneity not anticipated *a priori*.	Solutions vary across samples, variables, and methods; cluster membership rarely supplies a direct bedside treatment sequence.	A key validation benchmark for the GFM. Cluster models have stronger empirical grounding; the GFM is more explicitly action-oriented but remains hypothetical.	(14)
Gür Fibromyalgia Model	Five interacting, treatment-relevant domains weighted for current burden and sequencing.	Compact clinical logic for entry-point selection, readiness, and iterative reassessment.	Expert-derived, unvalidated, overlapping, and potentially reductive; predictive value is unknown.	Proposed as an operational hypothesis that complements rather than supersedes established frameworks.	(3–8, 14–32)
EULAR stepwise management	Education and non-pharmacological first-line care, followed by symptom-tailored options.	Evidence-based, widely used, and explicit about graduated management.	Provides less detail on how to choose among simultaneous barriers or set the first intervention intensity.	The GFM proposes a provisional within-guideline rule for ordering, readiness, and reassessment; superiority is untested.	(2)

The comparison is complementary rather than hierarchical. Established frameworks and data-driven clusters have stronger empirical support than the currently unvalidated GFM.

**Table 4:** Treatment-oriented comparison of major fibromyalgia frameworks and the specific sequencing hypothesis of the Gür fibromyalgia model.

Model/framework	Typical treatment logic	Where the framework is strongest	What remains unresolved	Specific GFM divergence	Key refs
Nociplastic/central-sensitization	Pain neuroscience education, centrally acting medication, graded exercise, and psychologically informed care.	Validates symptoms, supports mechanism-informed management, and helps avoid unnecessary structural investigation.	Does not consistently define which sustaining factor should be treated first.	Central amplification remains a target, but the initial sequence may instead be sleep-first, load-first, peripheral-first, or regulation-first.	(2–5, 33)
Biopsychosocial	Broad multimodal care using education, exercise, psychological support, behavioral change, and shared decisions.	Comprehensive whole-person care and therapeutic alliance.	May remain a parallel list of appropriate interventions without a rule for order, intensity, or readiness.	The GFM adds provisional weighting and entry-point logic, not a competing biopsychosocial theory.	(2, 4, 28, 30, 31, 51)
Stress-system/stress-disorder	Stress reduction, psychotherapy, autonomic downregulation, pacing, sleep support, and recovery-oriented care.	Particularly useful for marked reactivity, autonomic symptoms, volatility, and poor recovery.	May miss dominant biomechanical load, sleep pathology, or regional nociceptive input.	Regulation-focused care is used when that domain is dominant rather than assumed for all patients.	(24, 28, 29, 52)
Integrative neurophysiological–psychosocial	Individualized multimodal treatment combining biological and psychosocial interventions.	Strong systems-level rationale for multidomain care.	Clinical implementation can remain underspecified.	The GFM converts integration into a provisional sequence: identify the dominant barrier, stabilize it, then expand treatment.	(4–8, 28–32)
Mixed-pain/pain phenotyping	Mechanism-directed treatment of nociplastic, nociceptive, and neuropathic components.	Detects patients in whom a purely nociplastic pain description is incomplete.	Provides limited guidance on sleep restoration, pacing rhythm, and regulatory reserve.	Peripheral drivers are treated without equating the whole FM presentation with a mixed-pain phenotype.	(16, 22)
Data-driven clusters	Cluster-specific treatment hypotheses based on empirically observed subgroup profiles.	Offers the strongest route to empirically grounded stratification and discovery of treatment-effect heterogeneity.	Cluster solutions are not yet standardized and often lack a simple bedside allocation rule.	GFM sequences should be prospectively compared with cluster membership and outcomes rather than assumed to be superior.	(14)
Guideline-style multimodal care	Education, exercise, CBT-type strategies, selected medication, and self-management as evidence-based pillars.	Broad applicability and direct connection to guideline-supported interventions.	Often specifies what to offer more clearly than when to start, how rapidly to progress, or how to respond to low tolerance.	The GFM tests whether guideline-concordant care becomes more effective when ordered by current domain weight and readiness.	(2, 4–8, 26–31)
Gür Fibromyalgia Model	Start with the highest-yield domain, use low-burden early steps, then rebuild and expand with reassessment.	Explicit rationale for prioritization, timing, progression, and recalibration.	No prospective evidence yet that matching or sequencing improves outcomes.	Its distinguishing claim is testable: the order and intensity of otherwise familiar treatments may influence early trajectory.	(3–8, 16, 22–31)
EULAR stepwise algorithm	Begin with education and non-pharmacological care; add symptom-oriented treatment according to need.	Strong guideline foundation and broad European clinical relevance.	Does not fully specify tie-breaking when sleep, load, local pain, and recovery barriers are co-dominant.	GFM is proposed as a sequencing layer within EULAR-concordant care, not an alternative algorithm.	(2)

The GFM does not claim superior efficacy. Its incremental proposition is that treatment order and readiness can be prospectively tested alongside established multimodal care.

## 9 Research agenda and testable hypotheses

The long-term value of the GFM will ultimately depend on empirical testing. The aim of the research agenda outlined here is to move the model from conceptual plausibility to clinically informative validation.

An accessible first study would be a prospective cohort in which patients undergo baseline domain weighting and receive short-term phenotype-sensitive treatment allocation. The primary question would not be whether one phenotype is “true,” but whether domain-informed sequencing improves early treatment fit, adherence, symptom burden, or functional change.

Three initial hypotheses are stated formally. H1—Reliability: IF trained clinicians apply the provisional GFM checklist using a two-week recall window and explicit anchors, THEN inter-rater agreement for domain scores and the selected entry point will reach at least moderate reliability at baseline, BECAUSE structured anchors and tie-breaker rules reduce interpretive variability; the primary outcomes will be weighted kappa/intraclass correlation and entry-point agreement. H2—Construct validity: IF GFM profiles represent clinically meaningful but non-redundant patterns, THEN baseline domain scores will show prespecified convergent associations with relevant sleep, activity, pain, autonomic-recovery, and OMERACT measures while retaining discriminant separation at baseline, BECAUSE each domain is intended to capture a distinct treatment-relevant barrier; primary outcomes will be correlation and latent-profile/cluster concordance (14, 17). H3—Sequencing effectiveness: IF the first intervention is matched to the dominant or tie-breaker-selected domain, THEN patients will show greater adherence and treatment tolerance by 4–8 weeks and greater improvement in FIQR and function by 12–16 weeks than patients receiving guideline-concordant but non-sequenced multimodal care, BECAUSE lowering the principal barrier should improve readiness for subsequent treatment. A future trial should power its main outcome using a prespecified clinically meaningful change; the often-cited FIQ threshold is approximately 14% (about 8 points), while an FIQR-specific threshold should be justified for the selected population and design (49).

The five domains should be operationalized with pragmatic tools. Sleep can be tracked with diaries, the Pittsburgh Sleep Quality Index, the Insomnia Severity Index, or actigraphy; load imbalance with activity logs, step-count wearables, and pacing questionnaires such as the APQ-12 (50); peripheral drivers with focused musculoskeletal examination; regulation deficit with heart rate variability proxies, symptom-reactivity diaries, or stress-recovery measures; and central amplification with the Revised Fibromyalgia Impact Questionnaire, the Widespread Pain Index/Symptom Severity Scale, and pain drawings. Table 5 outlines a pragmatic operationalization strategy for research and bedside use.

**Table 5:** Practical operationalization of the five domains in the Gür fibromyalgia model.

Domain	Typical bedside clues	Candidate tools/measures	First therapeutic lever	Common pitfall
Central amplification	Widespread pain, hypersensitivity, disproportionate pain response	WPI/SSS, FIQR, pain drawing, symptom severity scales	Pain neuroscience education; tolerance-based graded rehabilitation	Reducing the whole syndrome to central sensitization alone
Sleep dysregulation	Unrefreshing sleep, morning worsening, cognitive fog, day-to-day variability	Sleep diary, PSQI, ISI, actigraphy when available	Sleep stabilization; CBT-I-informed strategies; treat coexisting sleep disorders	Treating sleep as secondary rather than mechanistically central
Load imbalance	Boom–bust cycle, post-exertional worsening, deconditioning, pacing failure	Activity diary, symptom log, step counts, brief pacing questionnaire	Recalibrate the load–recovery ratio; paced rehabilitation	Prescribing exercise too early or too aggressively
Peripheral drivers	Regional pain generators, myofascial burden, OA, obesity-related or hypermobility-related strain	Targeted musculoskeletal examination, pain map, local function testing	Reduce modifiable peripheral input without abandoning the FM frame	Endless structural searching or ignoring local drivers
Regulation deficit	Symptom volatility, stress reactivity, autonomic complaints, inconsistent routines	Recovery-time diary; orthostatic symptoms/vitals; Perceived Stress Scale; resting or challenge HRV (research adjunct only)	Stabilize routines; reduce autonomic/behavioral overload; CBT/ACT-informed pacing; evaluate orthostatic or sleep comorbidity	Psychologizing the syndrome or misreading it as simple non-adherence

ACT, acceptance and commitment therapy; CBT-I, cognitive behavioral therapy for insomnia; FIQR, revised fibromyalgia impact questionnaire; FM, fibromyalgia; HRV, heart rate variability; ISI, insomnia severity index; OA, osteoarthritis; PSQI, Pittsburgh sleep quality index; SSS, symptom severity scale; WPI, widespread pain index.

Table 6 extends this logic through a draft GFM checklist in which each domain is provisionally rated from 0 to 3. Its immediate purpose is research standardization rather than clinical diagnosis or mechanistic verification. Inter-rater reliability, construct validity, and responsiveness will need formal testing before any clinical adoption.

**Table 6:** Draft GFM checklist for provisional domain weighting in research settings.

Domain	Illustrative checklist item	Two-week severity anchors (0–3)	Suggested corroborating inputs
Central amplification	Is widespread pain, sensory hypersensitivity, or symptom intensity disproportionate to identifiable peripheral findings?	0: absent/background. 1: intermittent, mild, no material effect on plan. 2: frequent/clinically relevant and requires a treatment component. 3: dominant barrier that clearly determines the initial entry point.	WPI/SSS, FIQR, pain drawing, symptom-severity profile
Sleep dysregulation	Are unrefreshing sleep, sleep disruption, or morning worsening acting as major amplifiers of daytime symptom burden?	0: absent/background. 1: occasional poor sleep without consistent next-day effect. 2: frequent non-restorative/disrupted sleep with daytime amplification. 3: sleep failure repeatedly prevents engagement or drives major next-day worsening.	Sleep diary, PSQI, ISI, actigraphy when available
Load imbalance	Is the patient trapped in overload, underloading, or a boom-and-bust cycle in which demand repeatedly exceeds adaptive reserve?	0: demand and recovery broadly matched. 1: occasional mismatch, quickly corrected. 2: recurrent overload/underloading or boom–bust pattern affecting function. 3: demand-pattern mismatch is the main immediate barrier to progression.	Activity diary, step count/wearables, pacing log, APQ-12
Peripheral drivers	Are local nociceptive or biomechanical contributors meaningfully feeding total symptom burden?	0: no meaningful local contributor. 1: minor regional contributor. 2: modifiable local driver materially adds to burden. 3: a safe, clearly modifiable peripheral problem is the highest-yield initial target within confirmed FM.	Targeted musculoskeletal examination, pain map, local functional testing
Regulation deficit	Do ordinary perturbations produce disproportionate and prolonged difficulty in restoring autonomic, behavioral, and symptom equilibrium?	0: recovery proportionate. 1: occasional reactivity in one context. 2: recurrent cross-context volatility or delayed recovery. 3: generalized reactivity/recovery failure is the main barrier after sleep and urgent peripheral causes are addressed.	Symptom-reactivity diary, HRV proxy measures, orthostatic pattern, recovery-time tracking, routine consistency

APQ-12, 12-item activity pacing questionnaire; FIQR, revised fibromyalgia impact questionnaire; HRV, heart rate variability; ISI, insomnia severity index; PSQI, Pittsburgh sleep quality index; WPI/SSS, widespread pain index/symptom severity scale. Ratings refer to the previous 2 weeks. Severity and dominance are recorded separately in the research case-report form: the highest score is not automatically the entry point when safety, reversible comorbidity, treatment burden, or patient preference favors another domain. The scale and decision rules are provisional and require reliability and validity testing before clinical adoption.

Clinical trials should increasingly move beyond average-effect designs toward phenotype-sensitive approaches. The GFM offers a clinically interpretable framework for testing subgroup effects prospectively.

Digital phenotyping and ecological momentary assessment deserve dedicated evaluation because the GFM concerns changing domain weights rather than fixed labels. A feasible protocol could combine brief daily ratings of sleep quality, pain, fatigue, perceived stress, activity exposure, and recovery time with passive step count, sleep-wake timing, resting heart rate, and—where device quality permits—HRV. Within-person time-series analyses could test whether deterioration in one domain precedes changes in another and whether the selected entry intervention changes those temporal relationships. Digital measures should supplement, not replace, clinical assessment; device validity, missingness, burden, privacy, and algorithmic bias must be reported explicitly.

The framework also invites tighter integration of biological and behavioral research. Small-fiber pathology, neuroimmune findings, mitochondrial signals, and autonomic markers may become more clinically interpretable when they are linked to dominant domain constellations rather than studied in isolation (22–25).

## 10 Limitations

The GFM has important limitations. It is an expert-derived conceptual framework, not a validated classification or treatment-allocation system, and it has not been tested prospectively against treatment response. The purposive narrative method is susceptible to selection and confirmation bias, and no formal study-screening, risk-of-bias, or evidence-grading process was performed. Established diagnostic, mechanistic, guideline, OMERACT, and data-driven cluster frameworks have stronger empirical grounding. The five-domain structure was not derived by factor analysis, Delphi consensus, or cluster modelling; its simplicity may conceal within-domain heterogeneity and may not generalize across cultures, ages, sexes, comorbidity patterns, or care settings. Domain overlap is expected, tie-breaker rules are provisional, and the same patient may have several co-dominant entry points. Evidence is particularly limited for syndrome-wide benefit from treating peripheral drivers and for the specificity and clinical validity of physiological markers proposed for regulation deficit. Table 6 must therefore be used only as a research prototype until inter-rater reliability, construct validity, responsiveness, predictive validity, feasibility, and comparative clinical utility are established. A further limitation is the absence of formal patient and public involvement (PPI) in model development; domain definitions, assessment language, and sequencing priorities were derived by a single expert author without structured input from people living with fibromyalgia. Future iterations should incorporate co-design with patient partners to ensure that the framework reflects patient-valued outcomes, lived experience, and care priorities.

## 11 Conclusions

Fibromyalgia is poorly served either by reducing the syndrome to central amplification alone or by dispersing it into a loose inventory of symptoms and comorbidities. Without replacing established diagnostic, mechanistic, outcome, or clustering frameworks, the GFM proposes a middle-level clinical architecture in which central amplification, sleep dysregulation, load imbalance, peripheral drivers, and regulation deficit contribute in different proportions across patients and across time.

Its practical value lies in three proposed outputs: domain-weighted stratification after diagnosis, sequence-sensitive multimodal treatment planning, and a testable structure for prospective research. The intended shift is from generic multimodal care toward deliberate, sequence-sensitive multimodal care, while remaining within established diagnostic and guideline frameworks. Regulation deficit remains a provisional systems construct rather than a psychiatric label or biomarker-defined diagnosis, and peripheral drivers remain clinically relevant modifiers within—not outside—a confirmed nociplastic context. Acceptance of the model should depend on evidence that its domains can be scored reliably and that its sequencing rules improve adherence, tolerance, function, or quality of life beyond guideline-concordant usual care.

## Data Availability

The original contributions presented in the study are included in the article/Supplementary Material, further inquiries can be directed to the corresponding author.

## References

[1] Wolfe F Clauw DJ Fitzcharles MA Goldenberg DL Häuser W Katz RL. 2016 Revisions to the 2010/2011 fibromyalgia diagnostic criteria. Semin Arthritis Rheum. (2016) 46:319–29. 10.1016/j.semarthrit.2016.08.012 27916278

[2] Macfarlane GJ Kronisch C Dean LE Atzeni F Häuser W Fluß E. EULAR Revised recommendations for the management of fibromyalgia. Ann Rheum Dis. (2017) 76:318–28. 10.1136/annrheumdis-2016-209724 27377815

[3] Ablin JN. Nociplastic pain: a critical paradigm for multidisciplinary recognition and management. J Clin Med. (2024) 13:5741. 10.3390/jcm13195741 39407801 PMC11476668

[4] Jones EA Asaad F Patel N Jain E Abd-Elsayed A. Management of fibromyalgia: an update. Biomedicines. (2024) 12:1266. 10.3390/biomedicines12061266 38927473 PMC11201510

[5] Wu E Brener A Hiser JKD Hill DLK Farmer JO. Fibromyalgia: update on pathogenesis and management. Eur J Rheumatol. (2025) 12:0032. 10.5152/eurjrheum.2025.25032

[6] Jurado-Priego LN Cueto-Ureña C Ramírez-Expósito MJ Martínez-Martos JM. Fibromyalgia: a review of the pathophysiological mechanisms and multidisciplinary treatment strategies. Biomedicines. (2024) 12:1543. 10.3390/biomedicines12071543 39062116 PMC11275111

[7] Di Carlo M Bianchi B Salaffi F Atzeni F Govoni M Biasi G. Fibromyalgia: one year in review 2024. Clin Exp Rheumatol. (2024) 42:1141–9. 10.55563/clinexprheumatol/7bxyds 38607678

[8] Iannuccelli C Favretti M Dolcini G Gerardi MC Di Franco M Lucchino B. Fibromyalgia: one year in review 2025. Clin Exp Rheumatol. (2025) 43:957–69. 10.55563/clinexprheumatol/buhd2z 40470564

[9] D’Onghia M Ciaffi J Ruscitti P Cipriani P Giacomelli R Ablin JN. The economic burden of fibromyalgia: a systematic literature review. Semin Arthritis Rheum. (2022) 56:152060. 10.1016/j.semarthrit.2022.152060 35849890

[10] Amris K Ibsen R Duhn PH Lund Hetland M Bartels EM Dreyer L. Health inequities and societal costs for patients with fibromyalgia and their spouses: a Danish cohort study. RMD Open. (2024) 10:e003904. 10.1136/rmdopen-2023-003904 38307700 PMC10840036

[11] Lunn MPT Hughes RAC Wiffen PJ. Duloxetine for treating painful neuropathy, chronic pain or fibromyalgia. Cochrane Database Syst Rev. (2014) (1):CD007115. 10.1002/14651858.CD007115.pub3 PMC10711341 24385423

[12] Üçeyler N Sommer C Walitt B Häuser W. Anticonvulsants for fibromyalgia. Cochrane Database Syst Rev. (2013) (10):CD010782. 10.1002/14651858.CD010782 24129853

[13] Moore RA Derry S Aldington D Cole P Wiffen PJ. Amitriptyline for fibromyalgia in adults. Cochrane Database Syst Rev. (2019) 5:CD011824. 10.1002/14651858.CD011824 35658166 PMC6485478

[14] Gianlorenco AC Costa V Fabris-Moraes W Trevisan AC Battistella LR Barnabe C. Cluster analysis in fibromyalgia: a systematic review. Rheumatol Int. (2024) 44:2389–402. 10.1007/s00296-024-05697-1 38748219

[15] Yim YR Lee KE Park DJ Kim SH Nah SS Lee JH. Identifying fibromyalgia subgroups using cluster analysis: relationships with clinical variables. Eur J Pain. (2017) 21:374–84. 10.1002/ejp.935 27633925

[16] Saracoglu I Neblett R Akin E Basakci B Ayhan C Yilmaz F. Is fibromyalgia a nociplastic or a mixed-pain condition? International, multidisciplinary recommendations for pain phenotyping in fibromyalgia syndrome. Pain Physician. (2025) 28:483–94. 41337761

[17] Mease P Arnold LM Choy EH Clauw DJ Crofford LJ Glass JM. Fibromyalgia syndrome module at OMERACT 9: domain construct. J Rheumatol. (2009) 36:2318–29. 10.3899/jrheum.090367 19820221 PMC3419373

[18] Moldofsky H. Sleep and pain. Sleep Med Rev. (2001) 5:385–9. 10.1053/smrv.2001.0179 12531004

[19] Sivertsen B Lallukka T Petrie KJ Steingrímsdóttir ÓA Stubhaug A Nielsen CS. Sleep and pain sensitivity in adults. Pain. (2015) 156:1433–9. 10.1097/j.pain.0000000000000131 25915149

[20] Vincent A Benzo RP Whipple MO McAllister SJ Erwin PJ Saligan LN. Beyond pain in fibromyalgia: insights into the symptom of fatigue. Arthritis Res Ther. (2013) 15:221. 10.1186/ar4395 24289848 PMC3978642

[21] Schrepf A Kaplan CM Ichesco E Larkin T Bhavsar NA Murray T. Neurobiologic features of fibromyalgia are also present among rheumatoid arthritis patients. Arthritis Rheumatol. (2018) 70:611–8. 10.1002/art.40451 29439291

[22] Sommer C Üçeyler N. Small fiber pathology in fibromyalgia syndrome. Pain Rep. (2024) 10:e1220. 10.1097/PR9.0000000000001220 39726855 PMC11671067

[23] Findeisen K Guymer E Littlejohn G. Neuroinflammatory and immunological aspects of fibromyalgia. Brain Sci. (2025) 15:206. 10.3390/brainsci15020206 40002538 PMC11852494

[24] Di Bari A Demo G Gavio V Saracco M Rolla G Geuna S. Unravelling the relationship between anxiety, autonomic nervous system dysfunction and fibromyalgia: a systematic review. Clin Exp Rheumatol. (2025) 43:1136–45. 10.55563/clinexprheumatol/z1jcry 40556613

[25] Macchi C Giachi A Fichtner I Ferraro S Pucci B Molino Lova R. Mitochondrial function in patients affected with fibromyalgia syndrome is impaired and correlates with disease severity. Sci Rep. (2024) 14:30247. 10.1038/s41598-024-81416-z 39632893 PMC11618515

[26] Wang JJ Tam KW Hsiao HY Liou TH Rau CL Hsu TH. Effect of resistance exercises on function and pain in fibromyalgia: a systematic review and meta-analysis of randomized controlled trials. Am J Phys Med Rehabil. (2024) 103:275–83. 37535560 10.1097/PHM.0000000000002318

[27] de Souza LC Vilarino GT Andrade A. Effects of home-based exercise on the health of patients with fibromyalgia syndrome: a systematic review of randomized clinical trials. Disabil Rehabil. (2025) 47:80–91. 10.1080/09638288.2024.2329111 38588585

[28] Cojocaru CM Popa CO Schenk A Suciu BA Szasz S. Cognitive-behavioral therapy and acceptance and commitment therapy for anxiety and depression in patients with fibromyalgia: a systematic review and meta-analysis. Med Pharm Rep. (2024) 97:26–34. 38344332 10.15386/mpr-2661 PMC10852127

[29] Zhang R Li H Kong T Zhou B Tang D Gao L. The impact of community-based, common, non-pharmaceutical interventions on sleep in patients with fibromyalgia: a systematic review and network meta-analysis. Clin Exp Rheumatol. (2024) 42:2248–57. 10.55563/clinexprheumatol/53qrav 39051168

[30] Huang HX Apriliyasari RW Tsai PS. The effect of health education on symptom severity in patients with fibromyalgia: a systematic review and meta-analysis. Health Educ Res. (2025) 40:cyae035. 10.1093/her/cyae035 39485707

[31] Lebeau Foustoukos T Leclercq I Blanchard M Hügle T. Evidence-based self-management strategies for fibromyalgia: foundations for digital therapeutic applications. Interact J Med Res. (2026) 15:e67523. 10.2196/67523 41701966 PMC12957949

[32] Pinto AM Luís M Geenen R Castilho P Duarte C Marques A. Neurophysiological and psychosocial mechanisms of fibromyalgia: a comprehensive review and call for an integrative model. Neurosci Biobehav Rev. (2023) 151:105235. 10.1016/j.neubiorev.2023.105235 37207842

[33] Clauw DJ. From fibrositis to fibromyalgia to nociplastic pain: how rheumatology helped get US here and where do we go from here? Ann Rheum Dis. (2024) 83:1421–7. 10.1136/ard-2024-225656 39107083 PMC11503076

[34] Kosek E Cohen M Baron R Gebhart GF Mico JA Rice ASC. Do we need a third mechanistic descriptor for chronic pain states? Pain. (2016) 157:1382–6. 10.1097/j.pain.0000000000000507 26835783

[35] Bothelius K Kosek E Jansson-Frojmark M. In the end: associations between sleep disturbance and functional impairment in fibromyalgia—a path analysis study. J Sleep Res. (2025) 35:e70199. 10.1111/jsr.70199 40963006 PMC13193491

[36] Lederman S Arnold LM Vaughn B Engels JM Kelley M Sullivan GM. Pain relief by targeting nonrestorative sleep in fibromyalgia: a phase 3 randomized trial of bedtime sublingual cyclobenzaprine. Pain Med. (2026) 27:86–94. 10.1093/pm/pnaf089 40627411 PMC12773742

[37] Cohen H Neumann L Shore M Amir M Cassuto Y Buskila D. Autonomic dysfunction in patients with fibromyalgia: application of power spectral analysis of heart rate variability. Semin Arthritis Rheum. (2000) 29:217–27. 10.1016/S0049-0172(00)80010-4 10707990

[38] Lerma C Martinez A Ruiz N Vargas A Infante O Martinez-Lavin M. Nocturnal heart rate variability parameters as potential fibromyalgia biomarker: correlation with symptoms severity. Arthritis Res Ther. (2011) 13:R185. 10.1186/ar3513 22087605 PMC3334634

[39] Kivimäki AS Sallinen M Härmä M Salo P Lindholm H Hirvonen A. Heart rate variability and recovery from work-related stress in patients with fibromyalgia. Clin Auton Res. (2012) 22:245–52.

[40] McEwen BS. Protective and damaging effects of stress mediators. N Engl J Med. (1998) 338:171–9. 10.1056/NEJM199801153380307 9428819

[41] Smith MT Edwards RR McCann UD Haythornthwaite JA. The effects of sleep deprivation on pain inhibition and spontaneous pain in women. Sleep. (2007) 30:494–505. 10.1093/sleep/30.4.494 17520794

[42] Crombez G Vlaeyen JWS Heuts PHTG Lysens R. Pain-related fear is more disabling than pain itself: evidence on the role of pain-related fear in chronic back pain disability. Pain. (1999) 80:329–39. 10.1016/S0304-3959(98)00229-2 10204746

[43] Affaitati G Costantini R Fabrizio A Lapenna D Tafuri E Giamberardino MA. Effects of treatment of peripheral pain generators in fibromyalgia patients. Eur J Pain. (2011) 15:61–9. 10.1016/j.ejpain.2010.09.002 20889359

[44] Gür A Oktayoglu P. Advances in diagnostic and treatment options in patients with fibromyalgia syndrome. Open Access Rheumatol Res Rev. (2009) 1:193–209. 10.2147/OARRR.S8040 PMC5074721 27789991

[45] Eastwood F Godfrey E. The efficacy, acceptability and safety of acceptance and commitment therapy for fibromyalgia—a systematic review and meta-analysis. Br J Pain. (2024) 18:243–56. 10.1177/20494637241229549 38751564 PMC11092929

[46] Zhang Z Zhou P He N Liang Y Zhang Z Zhang Y. Patient version of guideline for fibromyalgia (2025 edition). J Evid Based Med. (2025) 18:e70094. 10.1111/jebm.70094 41410191 PMC12750489

[47] Pressimone C Lane K Ruppert K Engberg R Engberg S Brent LH. The development and implementation of a rheumatology referral-based, general internal medicine-led fibromyalgia clinic and preliminary patient outcomes. Musculoskeletal Care. (2024) 22:e1935. 10.1002/msc.1935 39261292 PMC11526333

[48] Turvill A Maratos F Sheffield D. Assessing the impact of interdisciplinary multimodal pain treatment on health-related quality of life in chronic pain: a systematic review and meta-analyses. Eur J Pain. (2026) 30:e70176. 10.1002/ejp.70176 41328667 PMC12670346

[49] Bennett RM Bushmakin AG Cappelleri JC Zlateva G Sadosky AB. Minimal clinically important difference in the fibromyalgia impact questionnaire. J Rheumatol. (2009) 36:1304–11. 10.3899/jrheum.081090 19369473

[50] Antcliff D McCray G MacKenzie R Marriott-Smith J Cawley K Holden MA. Testing the activity pacing questionnaire for validity, reliability and responsiveness: an outcome measure validation study. J Pain. (2025) 37:105568. 10.1016/j.jpain.2025.105568 41047057

[51] Turk DC Adams LM. Using a biopsychosocial perspective in the treatment of fibromyalgia patients. Pain Manag. (2016) 6:357–69. 10.2217/pmt-2016-0003 27301637

[52] Van Houdenhove B Egle UT. Fibromyalgia: a stress disorder? Piecing the biopsychosocial puzzle together. Psychother Psychosom. (2004) 73:267–75. 10.1159/000078843 15292624

